# Use of Exposure History to Identify Patterns of Immunity to Pneumonia in Bighorn Sheep (*Ovis canadensis*)

**DOI:** 10.1371/journal.pone.0061919

**Published:** 2013-04-26

**Authors:** Raina K. Plowright, Kezia Manlove, E. Frances Cassirer, Paul C. Cross, Thomas E. Besser, Peter J. Hudson

**Affiliations:** 1 Center for Infectious Disease Dynamics, Pennsylvania State University, University Park, Pennsylvania, United States of America; 2 Idaho Department of Fish and Game, St. Lewiston, Idaho, United States of America; 3 United States Geological Survey, Northern Rocky Mountain Science Center, Bozeman, Montana, United States of America; 4 Department of Veterinary Microbiology and Pathology, Washington State University, Pullman, Washington, United States of America; 5 The Huck Institute of Life Sciences, Pennsylvania State University, University Park, Pennsylvania, United States of America; Université de Sherbrooke, Canada

## Abstract

Individual host immune responses to infectious agents drive epidemic behavior and are therefore central to understanding and controlling infectious diseases. However, important features of individual immune responses, such as the strength and longevity of immunity, can be challenging to characterize, particularly if they cannot be replicated or controlled in captive environments. Our research on bighorn sheep pneumonia elucidates how individual bighorn sheep respond to infection with pneumonia pathogens by examining the relationship between exposure history and survival *in situ*. Pneumonia is a poorly understood disease that has impeded the recovery of bighorn sheep (*Ovis canadensis*) following their widespread extirpation in the 1900s. We analyzed the effects of pneumonia-exposure history on survival of 388 radio-collared adults and 753 ewe-lamb pairs. Results from Cox proportional hazards models suggested that surviving ewes develop protective immunity after exposure, but previous exposure in ewes does not protect their lambs during pneumonia outbreaks. Paradoxically, multiple exposures of ewes to pneumonia were associated with diminished survival of their offspring during pneumonia outbreaks. Although there was support for waning and boosting immunity in ewes, models with consistent immunizing exposure were similarly supported. Translocated animals that had not previously been exposed were more likely to die of pneumonia than residents. These results suggest that pneumonia in bighorn sheep can lead to aging populations of immune adults with limited recruitment. Recovery is unlikely to be enhanced by translocating naïve healthy animals into or near populations infected with pneumonia pathogens.

## Introduction

The population-level dynamics of infectious diseases in both time and space are shaped by individual-level responses to infection: how long an individual is infectious, how many individuals she or he infects, and how that host develops resistance to subsequent exposures. For example, a high R0 (basic reproductive rate of a disease) [1] coupled with lifelong immunity drives diseases like measles to become so-called childhood diseases characterized by an early age of infection and an adult population mostly resistant to infection but with a small proportion of susceptible individuals protected by herd immunity [2]. Infections with these characteristics can persist within populations larger than a critical community size, where births introduce a sufficient number of susceptible hosts to keep the effective R0 above unity [3], or by reinvasion of smaller populations within a metapopulation [4]. On the other hand, waning immunity, as observed with diseases such as whooping cough [5], [6], results in the reemergence of infections in older age cohorts [7], which in turn increases the likelihood of disease persistence and reduces the critical community size. If the immune response of the host is weak, then infections may persist within individuals, reducing condition and fitness; for example, helminths can produce persistent infections that reduce fecundity and generate oscillations in abundances of both parasites and hosts [8], [9].

Clearly, how the average individual responds to infection, and the variation in this response across the population, shapes population-level dynamics, and knowledge of these relations is essential for understanding and controlling infectious diseases [10]. However, elucidating individual-level responses to infection can be challenging, particularly when systems cannot be replicated in the laboratory and results of diagnostic tests are not correlated with resistance to infection or disease. Laboratory investigation of pneumonia in bighorn sheep (*Ovis canadensis*) has been challenging because secondary bacterial pneumonia masks the identity of the primary pathogen [11]. Recently, the bacterial pathogen *Mycoplasma ovipneumoniae* was identified as the most likely primary infectious agent [11]–[13]. *M. ovipneumoniae* impairs mucociliary clearance and increases the probability of multiple opportunistic lung infections that are the proximate cause of death [11]–[13].

**Figure 1:**
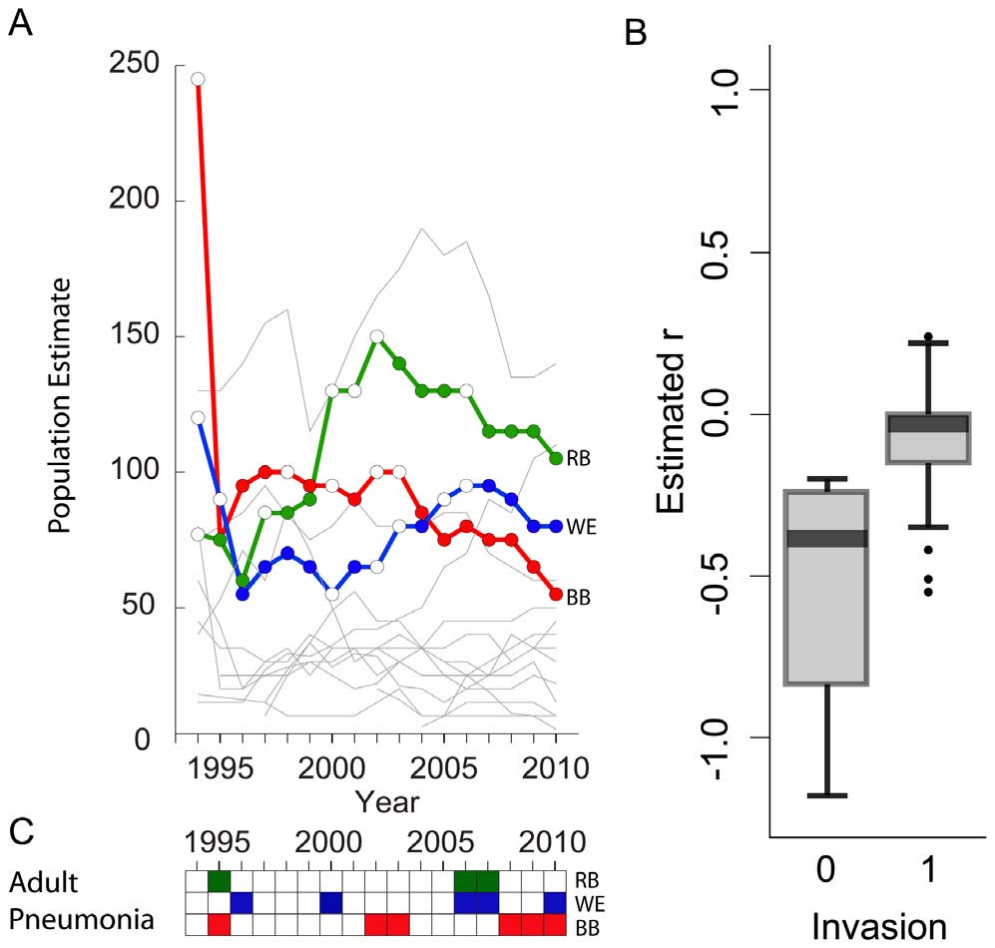
Pneumonia dynamics in Hells Canyon. A. Population estimates and pneumonia dynamics of monitored bighorn sheep populations in Hells Canyon (Idaho, Oregon, and Washington, U.S.A), 1994–2010. Colored lines represent the three most intensively monitored populations: Redbird (RB), Wenaha (WE), and Black Butte (BB). Opaque circles represent years with lamb pneumonia outbreaks (detected or suspected; see Cassirer et al. [20]), open circles represent years when no lamb pneumonia outbreak was detected or suspected. Grey lines represent population size estimates for all other populations monitored in Hells Canyon. The population estimates for Black Butte include the removal of 72 bighorn sheep during the 1995 epidemic [52]. B. Estimated population growth rate, r (natural log of population size in year t divided by population size in year t-1) during the year in which pneumonia invaded the population (invasion = 0) and in post invasion years with pneumonia mortalities in adults or lambs (invasion = 1). The invasion year r for Black Butte incorporates the removal of 72 bighorn sheep [52]. C. Years in which adult pneumonia mortality was detected in the three populations depicted in A: Redbird (RB), Wenaha (WE), and Black Butte (BB).

Confusion about the causative agent of pneumonia has constrained research on disease in bighorn sheep. Pneumonia in bighorn sheep continues to be one of the most poorly understood and intractable of the diseases that threaten wildlife in the United States and Canada. Moreover, despite substantial management efforts, ongoing mortality from pneumonia continues to impede the recovery of bighorn sheep since regional extirpation in many areas of the United States in the 1900s [14]–[16]. The effect of the disease during invasion (the first colonization of a population with pneumonia pathogens) is highly variable; infections of individuals in all age cohorts with up to 90% mortality are sometimes reported [17]; and events ranging from 30–50% mortality are commonly observed (Fig. 1a,b) [18]–[20]. Disease invasion frequently occurs during the breeding season (rut) in autumn and is followed by high adult mortality in autumn and winter [18]–[21]. After invasion, epidemics, manifested as summer pneumonia outbreaks in lambs prior to weaning, endure for a year to over a decade, whereas adult mortality from pneumonia is absent or low and sporadic (Fig. 1a,b) [14], [18], [20]–[23]. Bighorn sheep are spatially segregated by sex for most of the year [24]; ewes and lambs do not interact with mature rams or other sources of pathogens during summer. Moreover, candidate pneumonia agents are obligate parasites that do not persist in the environment; therefore, the assumption is that outbreaks in lambs originate from asymptomatic chronic carrier ewes [25]–[28]. Our premise was that the pattern of individual resistance to infection would reveal drivers of the population-level dynamics of pneumonia. Even in the absence of experimental immunological data, identifying these drivers could inform the development of management strategies to control the disease.

**Figure 2:**
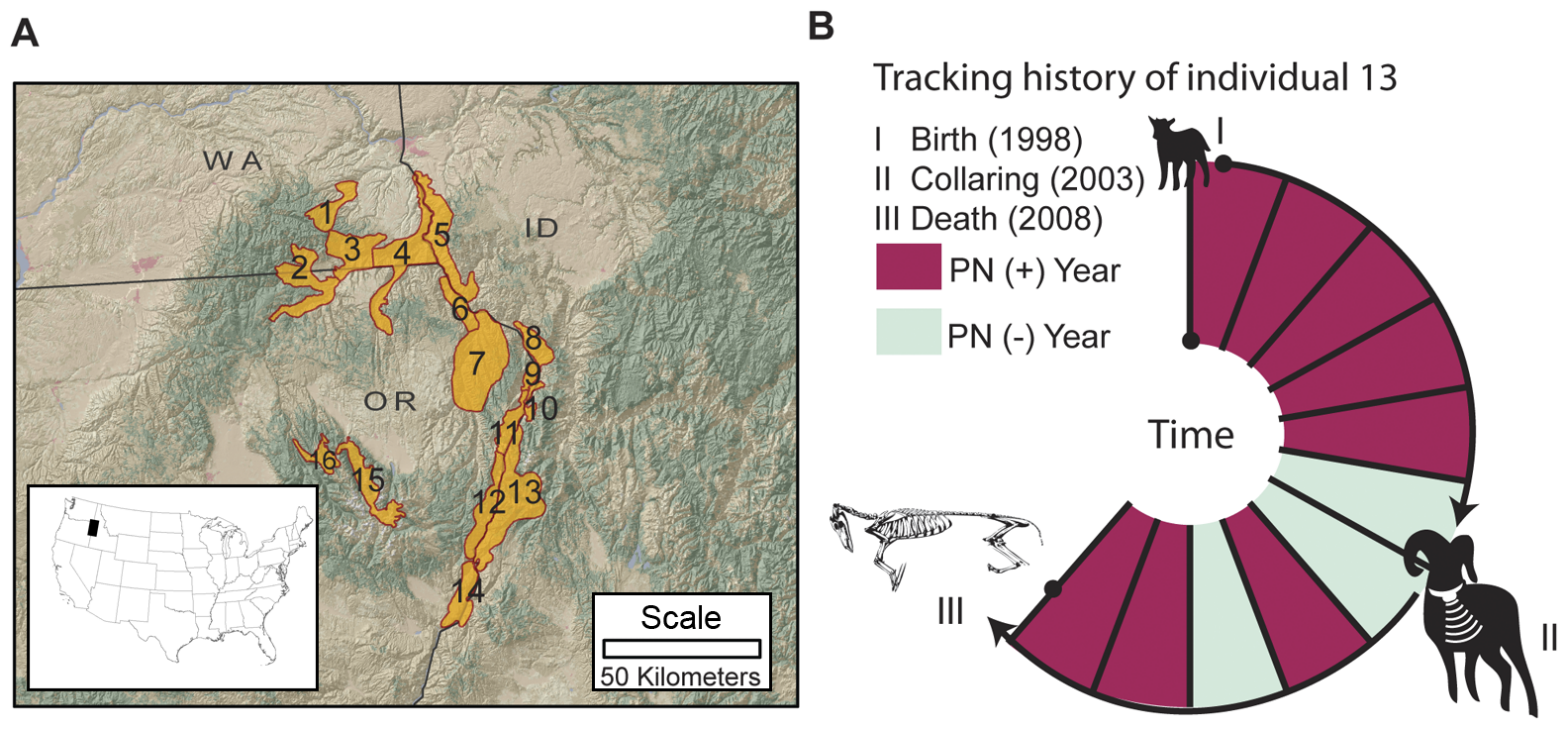
Study area and pneumonia history calculation. A. Study area: we report data from 388 radio-collared adult bighorn sheep and 753 ewe-lamb pairs within 12 of these 16 populations in Hells Canyon (WA = Washington, ID = Idaho, OR = Oregon; see Fig. S1 for populations' names and pneumonia histories). B. How individual pneumonia histories were constructed. The bighorn sheep in panel B was collared (II) at age 6 and died (III) at age 11 (in the middle of the biological year). Age was estimated at capture (II) or by incisor cementum analysis after death (III). Based on its population's pneumonia history (red indicates years with pneumonia mortality, green indicates years when pneumonia was not detected; see Fig. S1), this animal experienced 8 pneumonia exposures (*Count* = 8). The time since last exposure (*Lag*) was 0 when it died.

We examined individual-level responses to infection by analyzing disease-exposure history and pneumonia-induced mortality in 388 radio-collared bighorn sheep and 753 lambs born to 223 radio-collared ewes (Fig. 2a; Fig. S1) in 12 connected populations. At least 34 pneumonia epidemics occurred in these populations over a 14-year period, including invasion events that caused high mortality in all age cohorts and mortality events primarily restricted to lambs.

We developed alternative, but not mutually exclusive, hypotheses about the relationship between host immune response to infection and survival during subsequent exposures (Table 1). Each hypothesis was consistent with the observed dynamics: high adult mortality during pneumonia invasion, followed by low, sporadic adult mortality and frequent outbreaks of pneumonia in lambs. First, we hypothesized that a single exposure to pneumonia immunizes individuals against pneumonia during all subsequent exposures. Second, we hypothesized that immunity wanes in the absence of reexposure to disease. Third, we hypothesized that immunity is boosted by each exposure, so that the risk of dying decreases with increasing past exposures. Finally, we predicted that lambs born to previously exposed ewes are protected by maternally derived passive immunity.

**Table 1:** Potential relationships between past exposure of bighorn sheep to pneumonia and mortality during subsequent pneumonia epidemics.

Hypothesized relationship between infection (exposure) and immunity to disease	Predictions tested with models that included age as a baseline hazard and translocation status as a covariate
Exposure confers consistent long-term immunity	Risk of dying from pneumonia is highest during the first exposure and consistently low during subsequent exposures
Exposure confers immunity that wanes over time	Risk of dying from pneumonia is highest during the first exposure, surviving animals are protected for a short period of time and then their risk of dying when reexposed increases
Cumulative exposures strengthen immune response	Risk of dying from pneumonia decreases as number of exposures (*Count*) increases
Cumulative exposures strengthen immune response but immunity wanes between exposures	Risk of dying from pneumonia decreases as number of exposures (*Count*) increases but increases as time since exposure (*Lag*) increases
Exposure does not confer immunity	No relationship between risk of dying from pneumonia and any measures of past exposure
Exposure results in long-term infection	No relationship between risk of dying of pneumonia and measures of past exposure. Mortality is associated with specific risk factors for mortality in chronic carriers
Multiple exposures appear to strengthen immune response because weak or ‘frail’ individuals are most likely to die first	Risk of dying from pneumonia decreases as number of exposures (*Count*) increases
Ewes with more exposures transfer higher concentrations of immunoglobulins to lambs	Risk of lamb mortality decreases as maternal exposure increases

We assessed the relationship between previous exposure and survival by analyzing the relative risk of dying of pneumonia, conditional on an individual's pneumonia exposure history, including time since last exposure and number of past exposures (Fig. 2b; Fig. S1). We also analyzed the relationship between a ewe's exposure history and her lamb's survival. Our objectives were to obtain insights into responses of bighorn sheep to pneumonia, understand how resistance to infection affects population-level disease dynamics, and inform the assessment of management strategies such as supplementing populations with translocated animals and culling symptomatic individuals.

## Materials and Methods

### Study system and data

The Hells Canyon bighorn sheep study system includes 16 interconnected bighorn sheep populations containing approximately 800 animals. The populations occur over 23 thousand square kilometers in Idaho, Oregon, and Washington (U.S.A.; Fig. 2a). We report data from 388 radio-collared adults and 753 lambs born to 223 radio-collared ewes (Table 2) within 12 populations that were monitored through pneumonia epidemics from 1997 through 2010. Three of these populations were started with translocations from outside Hells Canyon during this study. The radio-collared animals represent a median of 24% of the adults in populations that range in size from less than 10 to more than 240 animals. We do not report data for radio-collared animals in populations that did not experience pneumonia epidemics (n = 51), or for animals for which we could not extrapolate an exposure history such as individuals translocated within Hells Canyon (n = 27), or animals from populations not regularly monitored during the study period (n = 11).

**Table 2:** Number of animals included in the analysis.

Radio-collared adults	Ewes	Rams	Total
Residents	196	110	**306**
Translocated	66	16	**82**
Total			**388**
**Outcomes and pneumonia-years**			
Died of pneumonia	32	15	**47**
Died of other causes or cause not determined	113	57	**170**
Censored	32	22	**54**
Still alive	85	32	**117**
Sheep-years	2586	761	**3347**
Sheep-pneumonia-years	1341	168	**1509**
Sheep-healthy-years	1245	593	**1838**
**Lambs**			
Total Lambs*			**753**
Lambs that died during lamb pneumonia outbreaks			**432**

* lambs born to radio-collared ewes with a known fate by October 1.

Animals were located at least every two weeks from the ground or air, and most of the more than 60,000 locations were visual observations. Survival, causes of mortality, movement, productivity, and whether a ewe's lamb survived to weaning were recorded for each radio-collared animal. Collared ewes and rams were followed for a maximum of 14.3 and 9.4 years, respectively. Data on population size and composition were collected in annual surveys. All animal capture and handling were conducted and coordinated by state wildlife agencies in accordance with accepted animal welfare protocols [29] (see Cassirer and Sinclair [14] and Cassirer et al. [20] for detailed field methodology).

Disease diagnoses were based on necropsies conducted at the Washington Animal Disease and Diagnostic Laboratory. A cause of death was determined for 173 radio-collared adults and 104 lambs that died during the study. Bacterial pneumonia was diagnosed in 47 (27%) of the adults and 92 (88%) of the lambs [20]. Difficulty finding freshly deceased unmarked lambs in relatively inaccessible terrain meant that some pneumonia outbreaks in lambs were inferred from observations of clinical signs and the distinct temporal signature of mortality associated with lamb pneumonia outbreaks [20]. Mortality from pneumonia occurred in at least one population every year during the study period, including at least three invasion events in populations of naïve translocated animals. Four populations had experienced invasion events in 1995 and 1996 before animals were radio-collared. Post-invasion dynamics were characterized by frequent outbreaks of pneumonia in lambs and sporadic low-level adult pneumonia mortality. Some populations experienced infrequent pulses of substantial adult pneumonia mortality, and all populations, excluding Sheep Mountain, experienced occasional healthy years (no pneumonia detected or suspected in adults or lambs) [20].

We used the results of the necropsies of adults and lambs, and field observations of pneumonia outbreaks in lambs, to classify the pneumonia status (healthy or pneumonic) of three classes of individuals, based on age and sex, within each population: ewes, rams, and lambs. We classified the pneumonia status of each of these classes within each population once each biological year, (defined as May 1–April 30, because most lambing occurs in May). We considered it reasonable to assign an annual disease status to each class because disease-related mortality is highly seasonal. Most lambs died June through August and most adults died October through February [14], [20]. Each class's annual pneumonia exposure status was binary: positive if there were pneumonia mortalities in that age or sex class within the biological year, and negative if there were no pneumonia mortalities within that class. Each individual's exposure history was then derived from the exposure history of its age or sex class within its population. For example, if ram(s) experienced pneumonia mortalities, we assumed that all surviving rams within that population were exposed. This assumption, that pneumonia mortality within a sex class results in exposure of all other animals of that sex, was based on the common observation that most adult mortality (sometimes up to 90% [17]) occurs in the year of pathogen invasion Therefore, most, if not all, members of a population must be exposed within that first year.

We differentiated pneumonia status by sex because sometimes mortalities occurred after the sexes had separated. We do not report results of models in which the sexes were aggregated, which yielded the same inferences as models in which the sexes were differentiated.

We also considered summer pneumonia events restricted to lambs as exposure events for ewes within that population. Lamb mortality rates were high (median 80%; putatively driven by high contact rates among lambs [20]). Intense lamb-ewe interaction likely exposes ewes to pneumonia-causing pathogens. Ewes never died of pneumonia during outbreaks in lambs from May through July [20], indicating protection (presumably immunity) from disease that probably was derived from previous exposure. Lamb-only pneumonia was not considered an exposure for rams because they have little-to-no contact with lambs or ewes during the summer pneumonia outbreaks in lambs [24].

We constructed a pneumonia-exposure history for each radio-collared adult on the basis of the pneumonia history of the age and sex classes within the population(s) of which it was a member (as described above; Fig. 2b, Fig. S1). We assumed that the population in which each animal occurred at the time of collaring was its natal population; if marked animals permanently dispersed to another population (rare within the data set) we adjusted their exposure status to reflect their known residence history. We based estimates of age, and thus exposure, prior to radio-collaring on horn annuli for rams [30], and on tooth eruption for ewes less than four years of age [30], [31]. We estimated the ages of ewes that died during the study on the basis of incisor cementum analysis [31] (n = 115). We assumed ewes with full adult dentition at capture were four years old when no incisors were available for aging (either the ewe did not die, or no incisors were collected at mortality). The longest exposure history (including the period from birth to radio-collaring) we constructed for a ewe was 19 years and for a ram was 13 years.

### Mortality hazard model construction

We characterized the relationship between pneumonia mortality and previous pneumonia exposure by fitting proportional hazards and logistic regression models implemented in the *survival* [32], *coxme* [33] and *lme4* [34] packages in R [35].

We used semi-parametric Cox proportional hazards models in which an individual's covariates changed over time [36] to assess whether previous exposure events changed an individual's relative risk of dying of pneumonia during an epidemic. These models estimate the effects of predictor variables on the response variable by comparing values of variables associated with individuals who died versus other individuals of the same sex and cohort (“risk set”). We grouped individuals into risk sets using two survival time-scales. First, we used a study-based timescale so that individuals were grouped by year, regardless of age. Second, we grouped individuals of the same age across years (Fig. S1) [37]. The former risk sets were small, especially early in the study, and we had limited power to detect trends in relative risk of mortality that were associated with any covariate except age. Furthermore, grouping individuals by age allowed us to incorporate age into the baseline hazard of dying while explicitly estimating the effects of other covariates; we therefore report results from the age scale.

Our models had four fixed effects: translocation status (*Source*; a binary variable set at 1 if an individual was translocated and 0 if it was resident); whether an individual previously was exposed to pneumonia (*IPrevious*; a binary variable); the number of previous exposure events (*Count*; the number of biological years with confirmed pneumonia within the individuals' population of residence); and the number of years since the most recent exposure event (*Lag*). Our sample size was insufficient to examine interaction effects.

The saturated model of the *i^th^* individual's hazard of dying of pneumonia at age *a*, *h_i_*(*a_i_*|*β_i_*), was a function of the baseline hazard at age *a*, *h_0_*(*a_i_*), as well as a linear combination of the covariates:
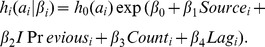



We did not include population as a source of shared frailty because in some populations *Count* or *Lag* was identical for all individuals within a risk set over successive years, preventing within-population estimation of the covariate effects. For a subset of ewes born during the study period that were aged by cementum analysis, we also examined the effect of pneumonia status of lambs during their birth year on probability of mortality.

We evaluated models that included all combinations of the covariates described above, with the exception of *IPrevious* and *Lag*, which were identical for individuals with no past exposure to pneumonia. We examined scaled Schoenfeld residuals as a function of time for all fitted models to assess whether the proportional hazards assumption was met. We used standard metrics to examine whether the models included overly influential points, and assessed Martingale residuals to check whether variance was constant across values of all covariates. Models without higher-order terms or shared frailty components had no overly influential points and met the proportional hazards assumption of consistent relative risk across time. Statistical significance was assessed at α = 0.05.

### Maternal analysis

We fit both proportional hazards and logistic regression models to examine whether maternal exposure history was associated with either the timing or the rate of lamb mortality prior to weaning. To monitor lamb survival to weaning we identified lambs born to radio-collared ewes through observations of close association and suckling. Ewes were observed weekly during lambing to determine whether or not they produced a lamb. We attempted to locate ewes with lambs at least weekly during lactation and all radio-collared ewes were observed a minimum of every two weeks during this period. We assumed lamb mortality had occurred if the radio-collared ewe was no longer associating with the lamb prior to the expected date of weaning (October 1) [20], [38]. We examined data from 753 lambs born to 223 radio-collared ewes (ewes almost always give birth to a single lamb) over 14 years. Of these lambs, 432 were born in years with pneumonia outbreaks in lambs and 321 were born in years without pneumonia (detected or suspected).

Within the proportional hazards models of lamb mortality, we accounted for the effect of a given ewe on the relative risk of dying by including a shared frailty term (*Ewe*) for all lambs born to the same ewe [39]. We also included four fixed effects: count of ewe's previous exposure events (*Count*); ewe translocation status (*Source*); estimated ewe age (*EweAge*); and whether the lamb was born in a pneumonia year (*PneuYear*).

The saturated model of the mortality risk for the *j^th^* lamb born to the *i^th^* ewe at time *t*, *h_ij_*(*t_ij_*|*β_i_*), relative to the baseline hazard (*h_0_*(*t_ij_*)), is:
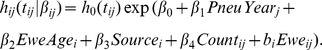



We did not include *Lag* in the lamb-mortality models because this would require that some ewes have *Lag*>0. This would conflict with our assumption that carrier ewes are the source of outbreaks in lambs: we assume that the ewe population must be infected immediately before the lamb population, because ewes serve as the source of lamb infection.

We used the same ewe-level covariates defined above, including the random effect *Ewe*, in logistic regression models to assess the effect of ewe-exposure history on lamb mortality through October 1st. In addition, we investigated trade-offs between reproduction and immunity with a Cox proportional hazards model to assess the risk of ewe pneumonia mortality given the survival or death of her previous year's lamb. We hypothesized that death of a lamb in year *t-1* could increase the probability that its mother survived a pneumonia epidemic in year *t*.

## Results

### Ewes

**Figure 3:**
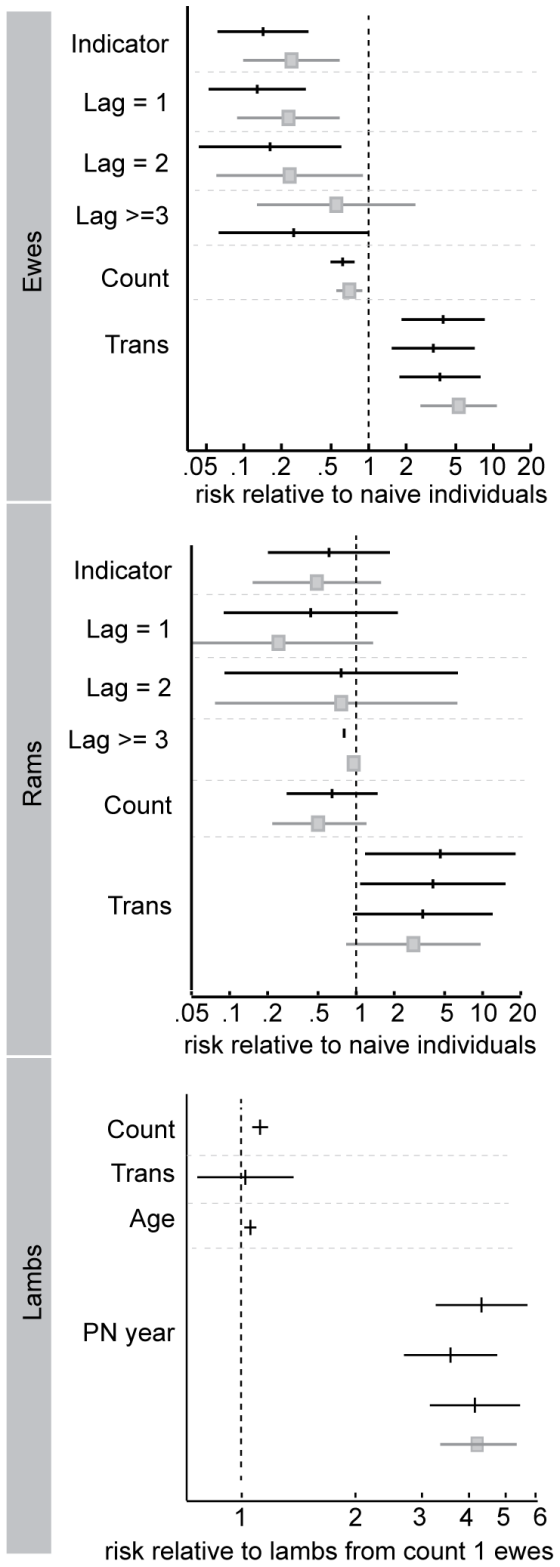
Cox proportional hazards model-estimated relative risks of mortality of ewes, rams, and lambs after accounting for differences in sex and age. Risk of dying of pneumonia relative to naïve individuals given: an indicator for any previous pneumonia exposure (*Indicator*), the number of years since the last exposure event (*Lag* of 1, 2 or > = 3), increasing number of exposure events (*Count*), and translocation status (*Trans*). Bottom panel: model-estimated risk of dying for lambs during outbreaks of pneumonia given ewe covariates. Coefficient estimates from univariate models are in grey and coefficient estimates from multivariate models are in black. Risk values are drawn from proportional hazards model coefficient estimates, with the point estimate denoted by the box or vertical line, and horizontal lines extending to the 95% confidence limits.

Our analyses showed that translocation status was the covariate most strongly associated with the probability of dying of pneumonia. Translocated ewes' risk of dying of pneumonia was about three times greater than that of residents' (Fig. 3; Table 3). Translocation did not have a statistically significant effect on mortality risk in years without pneumonia epidemics (Table 4). The statistical significance and relative change in risk associated with translocation was similar among all ewe models.

**Table 3:** Ewes: results from Cox proportional hazards model of ewe relative risk of dying from pneumonia given covariates over the age-based timescale.

Model	Covariate	Beta	Exp. Beta (95% CI)	SE	P-value	AIC	Delta AIC
**Count & translocation**	Count	−0.36	0.70(0.55, 0.89)	0.12	0.002	245.15	0.17
	Translocated	1.19	3.32(1.54, 7.13)	0.39	0.004		
**Past exposure & translocation**	Exposed	−1.43	0.26 (0.10, 0.58)	0.45	0.002	244.98	0
	Translocated	1.32	3.76(1.78, 7.95)	0.38	0.0005		
**Lag & translocation**	Lag 1 Yr	−1.53	0.22 (0.08, 0.55)	0.48	0.001	246.45	
	Lag >/ = 2 Yr	−1.16	0.31(0.10, 0.96)	0.57	0.041		1.47
	Translocated	1.31	3.72(1.76, 7.86)	0.38	0.0006		
**Lag (4 categories) & translocation**	Lag 1 Yr	−1.49	0.22(0.09, 0.58)	0.48	0.0003	247.43	2.45
	Lag 2 Yr	−1.46	0.23(0.06, 0.89)	0.70	0.034		
	Lag > = 3 Yrs	−0.60	0.55(0.13, 2.35)	0.74	0.417		
	Translocated	1.38	3.98 (1.85, 8.57)	0.39	0.0004		
**Translocation**	Translocated	1.67	5.30(2.62,10.73)	0.36	<.0001	252.18	7.2
**Count**	Count	−0.48	0.62(0.49, 0.77)	0.11	<.0001	252.45	7.47
**Past exposure**	Exposed	−1.95	0.14(0.06, 0.32)	0.43	<0.0001	254.56	9.58
**Lag**	Lag 1 Yr	−2.08	0.13 (0.05, 0.31)	0.46	<.0001	255.83	10.85
	Lag >/ = 2 Yr	−1.64	0.19(0.07, 0.56)	0.54	0.0026		

SE = Standard error.

**Table 4:** Ewes in non-pneumonia (healthy) years: impact of covariates on the relative risk of dying (of causes other than pneumonia) outside of pneumonia epidemics.

Model	Covariate	Beta	Exp. Beta (95% CI)	SE	P-value	AIC	Delta AIC
**Count & Translocation**	Count	−0.042	0.95 (0.76, 1.21)	0.12	0.62	217.64	1.76
	Translocated	0.225	1.25 (0.51, 3.06)	0.46	0.72		
**Past exposure & translocation**	Exposed	0.35	1.42 (0.51,3.92)	0.52	0.50	217.30	1.42
	Translocated	0.39	1.48 (0.60, 3.63)	0.46	0.40		
**Lag & translocation** *	Lag 1 Yr	0.39	1.47 (0.52, 4.17)	0.53	0.46	219.59	3.71
	Lag 2 Yrs	0.64	1.89 (0.53, 6.70)	0.64	0.33		
	Lag > = 3 Yrs	−0.62	0.54 (0.06, 4.85)	1.12	0.58		
	Translocated	0.39	1.47 (0.60, 3.64)	0.46	0.40		
**Count**	Count	−0.06	0.94 (0.76, 1.17)	0.11	0.58	215.88	0
**Past exposure**	Exposed	0.21	1.23 (0.48, 3.17)	0.48	0.67	216.00	0.12
**Lag**	Lag 1 Yr	0.26	1.29 (0.48, 3.46)	0.50	0.61	218.27	2.39
	Lag 2 Yr	0.47	1.60 (0.48, 5.25)	0.61	0.44		
	Lag > = 3 Yr	−0.78	0.46 (0.05, 3.92)	1.10	0.47		

SE = Standard error.

* Model was inestimable, since all pneumonia-year mortalities among individuals with lags of 2 or more occurred among residents; the translocation coefficient could not be calculated.

Past exposure was significantly associated with a decrease in relative risk of pneumonia, as were the number of previous exposure events (*Count*). The relative risk of pneumonia mortality increased with time since last exposure (*Lag*). The direction of these covariates suggests that previous exposure confers immunity. The change in values and statistical significance of *Count* and *Lag* beyond the first year suggests that waning and boosting may modulate the level of immunity. AIC values based on partial likelihoods were similar among all multivariate models (Table 3). Even if all hypotheses were correct, individuals' immunity could both decrease and increase over their lifetimes with waning and boosting, respectively. However, the comparable level of support for all three models indicated that none of our hypotheses could be rejected (Table 1). As previously noted, the hypotheses are not mutually exclusive, and the data are not likely to fully discriminate between them. Thus, we did not focus on the relative support for the various hypotheses. Instead, we relied on the estimated effect of each covariate on an individual's hazard of dying of pneumonia to gain insight into individuals' immune responses.

**Figure 4:**
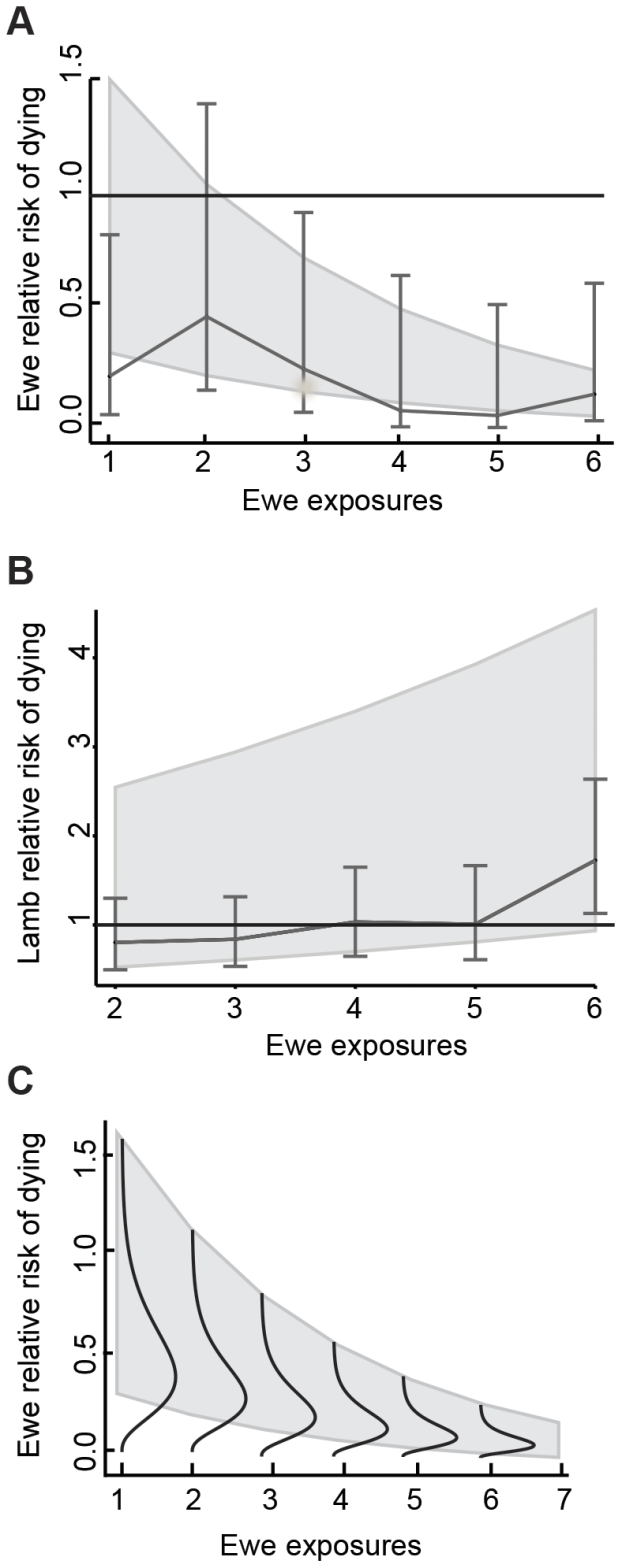
Relationship between cumulative past exposure and risk of dying. a. Ewe risk of dying from pneumonia as a function of the number (*count*) of previous exposures (relative to unexposed ewes). A relative risk of 1 on the y-axis represents no effect of *count* on the risk of dying of pneumonia. The shaded area represents the 95% confidence bounds on the hazard ratio associated with continuously increasing number of previous exposures (in a model fit on an age-scale that included a fixed effect for translocation status). The error bars bound the 95% confidence range for the uniquely estimated hazard ratios associated with each value of *count*. The line connects the estimated median risk of dying relative to the risk for previously unexposed ewes. *Count* values above six are grouped within the *count* = 6 category. b. Lambs' risk of dying in a pneumonia epidemic given the number of times the lamb's mother was exposed. The shaded area represents the 95% confidence bounds on the hazard ratio associated with continuously increasing number of previous exposures (*count*) of the ewe (in a model fit on lamb age-scale that included a fixed effect for ewe's translocation status). The error bars bound the 95% confidence range for the uniquely estimated hazard ratios associated with each number of exposures for ewes. The line connects the estimated median risk of lamb mortality. c. A conceptual diagram illustrating how individual frailty may drive the apparent relationship in part a. Frailties decrease as number of exposures (or age) increase because the weak (at the upper tail of the distribution) die first, and cannot be observed in later years, leaving an increasing proportion of strong individuals.

The negative coefficient on *Count* suggests that a ewe's relative risk of pneumonia mortality decreases slightly as the number of past exposure events increases (Figs. 3,4*a*). Time since previous exposure (*Lag*) was statistically significantly associated with changing mortality risk. The risk that previously exposed ewes would die from pneumonia was approximately 22% (95% confidence interval 0.09, 0.58) of that of naïve (unexposed) ewes for two years after exposure. The risk of dying of pneumonia three or more years after exposure was not significantly different from the risk of a naïve individual, suggesting that protective immunity may wane after two-to-three years (Table 3). However, sample size of ewes with *Lag*>2 was very small. Ewes of known age (cementum-aged) that were born during an outbreak of pneumonia in lambs and survived did not have higher or lower probability of dying compared to ewes of known age born in a year with no pneumonia detected (Table S1). None of the models explained mortality risk in years without pneumonia (Table 4).

### Rams

Translocation status was the only covariate with a significant effect on mortality risk in rams. The risk that translocated animals would die of pneumonia was around 4 (95% confidence interval 1.10, 15.14) times that of resident rams in a model including *Count* and 4.50 (95% confidence interval 1.20,17.19) times that of residents in a model including *Lag* (Fig. 3; Table 5). *Count* and *Lag* had negative coefficients, but were not statistically significant. Support for all multivariate models was similar on the basis of AIC values (Fig. 3; Table 5).

**Table 5:** Rams: results from Cox proportional hazards model of ram hazard of dying from pneumonia given covariates over the age-based timescale.

Model	Covariate	Beta	Exp. Beta (95% CI)	SE	P-value		AIC	Delta AIC
**Translocation**	Translocated	1.04	2.83 (0.83, 9.60)	0.62	0.10		78.5	0.6
**Count & Translocation**	Count	−0.67	0.51 (0.22, 1.21)	0.44	0.13	77.9		0
	Translocated	1.40	4.04 (1.10, 5.14)	0.67	0.04			
**Past exposure & translocation**	Exposed	−0.72	0.49 (0.15, 1.57)	.60	.23		79.1	1.2
	Translocated	1.21	3.36 (0.94, 11.99)	0.65	0.06			
**Lag & translocation**	Lag 1 Yr	−1.40	0.25 (0.04,1.37)	0.87	0.11		79.4	1.5
	Lag >/ = 2 Yr	−0.16	0.85 (0.21, 3.44)	0.71	0.83			
	Translocated	1.50	4.50 (1.20, 17.19)	0.68	0.03			
**Count**	Count	−0.44	0.65 (0.28, 1.48)	0.42	0.30		80.0	2.1
**Past exposure**	Exposed	−0.49	0.61 (0.20, 1.85)	0.57	0.38		80.4	2.5
**Lag**	Lag 1 Yr	−0.82	0.44 (0.09, 2.13)	0.81	0.31		82.0	4.1
	Lag >/ = 2 Yr	−0.24	0.79 (0.21, 2.96)	0.67	0.73			

SE = Standard error.

### Lambs

In all models, the relative risk of lamb death prior to weaning in years with pneumonia outbreaks was approximately four times that in years without outbreaks (Fig. 3; Table 6). The exponentiated ewe-frailty terms ranged from 0.83 to 1.16, suggesting that the median probability of lamb mortality increased by a maximum of 16% for the worst-performing ewe and decreased by a maximum of 17% for the best-performing ewe (in a model including pneumonia years and healthy years). Ewe-translocation status was not reliably associated with altered lamb mortality risk (Fig. 3; Table 6).

**Table 6:** Lambs: results from Cox proportional hazards models of lamb hazard of dying given dam (ewe) covariates in all years (pneumonia and healthy years; top), years without pneumonia (pneumonia years excluded; middle) and years with pneumonia (healthy years excluded; bottom).

Model	Covariate	Beta	Exp. Beta (95% CI)	SE	P-value	SD of ewe shared frailties	AIC	Delta AIC
**Lambs in all years**								
**Ewe Only**	PN Year	1.46	4.30 (3.27, 5.64)	0.14	<.0001	0.21	4411.43	18.7
**Count**	PN Year	1.27	4.17 (2.68, 4.73)	0.15	<.0001	0.21	4392.73	0
	Count	0.11	1.12 (1.07, 1.17)	0.02	<.0001			
**Translocation**	PN Year	1.46	4.32 (3.26, 5.71)	0.14	<.0001	0.21	4413.41	20.68
	Translocated	0.02	1.02 (0.76, 1.37)	0.15	0.88			
**Age**	PN Year	1.42	4.15 (3.15, 5.46)	0.14	<.0001	0.24	4405.60	12.87
	Age	0.05	1.05 (1.02, 1.09)	0.02	0.005			
**Age & Count**	PN Year	1.28	3.59 (2.70, 4.78)	0.15	<.0001	0.22	4394.44	1.71
	Age	0.01	1.01 (0.97, 1.06)	0.02	0.59			
	Count	0.10	1.11 (1.05, 1.17)	0.03	<.0001			
**Trans & Count**	PN Year	1.28	3.66 (2.75, 4.88)	0.15	<.0001	0.18	4393.56	0.83
	Count	0.12	1.12 (1.07, 1.18)	0.02	<.0001			
	Translocated	0.17	1.19 (0.88, 1.60)	0.17	0.26			
**Lambs in years without pneumonia**								
**Ewe only**	Ewe					0.20	722.81	0
**Translocation**	Translocated	−0.35	0.70 (0.40, 1.22)	0.28	0.21	0.02	723.17	0.36
**Count**	Count	0.06	1.06 (0.95, 1.19)	0.06	0.29	0.02	723.17	0.36
**Age**	Age	−0.01	0.99 (0.91, 1.09)	0.05	0.87	0.02	724.72	1.91
**Age & Count**	Age	−0.05	0.96 (0.85, 1.07)	0.06	0.40	0.02	724.98	2.17
	Count	0.10	1.10 (0.96, 1.27)	0.07	0.18			
**Lambs in years with pneumonia**								
**Ewe only**	Ewes					0.267	3342.74	19.51
**Translocation**	Translocated	0.19	1.20 (0.86, 1.69)	0.17	0.28	0.242	3343.66	20.43
**Count**	Count	0.13	1.14 (1.08, 1.20)	0.03	<.0001	0.279	3323.23	0
**Age**	Age	0.07	1.07 (1.03, 1.11)	0.02	0.001	0.308	3334.46	11.23
**Age & Count**	Age	0.02	1.02 (0.98, 1.07)	0.02	0.34	0.276	3324.32	1.09
	Count	0.11	1.12 (1.05, 1.19)	0.03	0.001			

SE = Standard error; SD = standard deviation; PN Year = years with outbreak of pneumonia in lambs.

Paradoxically, a lamb's risk of dying significantly increased with its mother's previous exposures during years with pneumonia outbreaks (Fig. 4*b*; Table 6), but not during years without outbreaks (Table 6). The number of previous exposures and ewe age were collinear; however, number of previous exposures (but not age) was statistically significantly associated with risk of mortality in a model that included both covariates (Table 6).

There were no naïve dams (mothers) during lamb epidemics. Hence, we compared each value of *Count* to a baseline of *Count* = 1 (one previous exposure to pneumonia); a greater effect might be expected if *Count* = 0 was the baseline. We did not find a significant relation between the mortality risk of a ewe during exposure to pneumonia in year t and the survival or mortality of her lamb in the year t-1. Results from the logistic regression models were consistent with the results from the proportional hazard models (Table S2).

## Discussion

We used data on host survival to draw inferences about immunological processes in a system where the etiological agent is unknown and thus serology-based inferences are not feasible. We examined whether previous exposure protects bighorn sheep from pneumonia and whether the strength of the response (presumably immunity) is a positive function of the number of previous exposures to pneumonia and a negative function of time since exposure. We also explored whether passively acquired immunity protects offspring during lamb pneumonia outbreaks. Our results indicate that past exposure decreases ewes' risk of dying from pneumonia. More-frequent exposure of ewes to pneumonia was associated with higher offspring mortality during outbreaks of pneumonia. We were unable to discern the specific dynamics of immunity in ewes because models with waning, boosting, or consistent immunity were similarly supported. Furthermore, epidemiological processes such as herd immunity and individual frailty may generate patterns analogous to waning and boosting immunity, respectively.

### Time since exposure (Lag) and number of exposures (Count)

Waning immune responses are consistent with our understanding of upper- and lower-respiratory tract immunity. Waning immunity may be a consequence of antigenic variation, immune system hyporesponsiveness (induced by commensal flora involved in secondary pneumonia) [40], [41], or immune exclusion (secretory IgA binding to bacterial pathogens and preventing development of adaptive immunity) [41]. However, two aspects of the data prevented us from differentiating waning immunity from consistent immunizing exposure (risk of dying consistent over time since exposure). First, we documented few fade-out events (years without observed pneumonia); and, second, fade-out events were of short duration. Therefore, sample sizes for investigating waning immunity were limited and immune boosting from frequent exposure likely masked waning. Furthermore, herd immunity (proportion of immune animals in the population) inherently confounds the effects of time since exposure on immunity; while disease is absent, recruited susceptible juveniles gradually dilute the pool of immune animals, hence herd immunity declines even if individual immunity remains constant. Therefore, the risk of exposure (and subsequent disease) increases with time since an epidemic. Finally, survival probability declines in older animals [19] and therefore age may confound the relationship between survival and time since exposure, particularly if we underestimated the ages incorporated into the baseline hazard.

The data suggest a trend of decreasing mortality with increasing exposures (*Count*) to pneumonia. At least two phenomena may account for this effect. First, immunity may be dose-dependent: each successive exposure may strengthen the anamnestic immune response (immune memory) to a particular agent, or diversify exposure to multiple primary and secondary agents. Second, inherently weak or high-risk individuals (for example, individuals with weaker innate immune responses or highly social individuals) are more likely to die first (individual frailty) [42], [43] when their exposure counts are, coincidently, lower. Increasing proportions of stronger individuals remaining in the population are exposed repeatedly, generating an apparent relationship between number of exposures and risk of mortality (Fig. 4*c*). The removal of age through its incorporation into the baseline hazard, and the observed relationship of increasing mortality as a function of age (Fig. S2), suggest that age is not driving this relationship. The data did not allow an examination of cumulative exposure within each level of *Lag*.

### Lamb survival and maternal immunity

We had hypothesized that ewes with more exposures would transfer higher concentrations of passively acquired immunoglobulins to their lambs, resulting in lower lamb mortality. By contrast, the data showed that increasing ewe exposures were weakly associated with earlier and higher lamb mortality. This relation was opposite to the relationship between number of exposures of ewes and ewe mortality. The earlier timing of lamb death for ewes with more exposures suggests that the force of infection to lambs varies among mothers with differing exposure histories. One potential explanation is that ewes with more exposures are more likely to be infectious carriers (perhaps either cumulative exposures or age increase the risk of becoming a carrier), providing direct and early exposure to their lambs.

We considered reproductive senescence as an alternative explanation, because number of exposures and age are inherently collinear. However, the relationship between number of ewe exposures and earlier or higher lamb mortality was only observed during pneumonia epidemics (although a paucity of ewes with high numbers of exposures in years without pneumonia made assessment difficult); furthermore, Festa-Bianchet and King [38] showed no difference in lamb mortality between prime-age and older bighorn ewes in pneumonia-free populations. Variations in pathogen virulence or the number of carrier ewes over time are alternative explanations.

We assumed all ewes that gave birth to lambs during pneumonia epidemics had prior exposure to pneumonia; therefore, we could not examine the effect of presence versus absence of passively transferred maternal immunity on lamb mortality. Given the extremely high lamb mortality rates during pneumonia outbreaks among lambs in populations with previous exposures, it appears that passive immunity transferred from the ewe does not prevent lamb mortality. Besser et al. 's [13] detection of pulmonary *M. ovipneumoniae* infection in asymptomatic lambs as young as four days old, and the development of bronchopneumonia in (passively) seropositive 10 day old lambs, similarly suggests that passive immunity has little effect in delaying progression of pneumonic disease. Given the inverse relationship between number of previous exposures of ewes and timing of lamb death discussed above, ewe infection status (leading to early lamb exposure) may be a better predictor of lamb mortality than the ewe's maternal antibody concentration (presuming that multiple exposures increase maternal antibody concentration).

### Rams

We did not find an association between past exposure to pneumonia and ram mortality. However, the few rams in this study limited our ability to examine the association of exposure covariates with mortality. Furthermore, ram exposure is difficult to monitor. Rams are more likely to be exposed through unobserved interactions with other bighorn sheep populations and domestic sheep populations, particularly during the rut. Rams are also spatially separated from ewe-lamb groups so lamb pneumonia cannot be used as a sentinel of disease transmission, potentially leading to underestimation of exposure. On the other hand, the lack the reexposure opportunities, due to separation from summer lamb pneumonia outbreaks, could drive real differences in exposure patterns between rams and ewes. Also, sexual dimorphism in immune function is well documented in some species [44], [45] and factors such as the immunosuppressive effects of testosterone, and life history differences between sexes, could be responsible for different responses to disease exposure.

### Translocation

Even when we accounted for previous exposure, number of previous exposures, time since previous exposures, and age and sex, translocated animals had three-to-four times the risk of dying of pneumonia of resident animals, a result consistent with previous studies [26], [46]. Translocated animals did not enter the study until the biological year following translocation (2–4 months after release), pneumonia deaths occurred from 2–5 years after release, and animals translocated into populations without pneumonia did not die of pneumonia. Therefore, it is unlikely that the act of translocating, or short-term post-release effects such as stress, contributed to the higher risk of dying of pneumonia. We suspect that two issues account for the difference between translocated and resident animals. First, these data did not capture the major invasion events, and associated high mortality, that occurred in naïve resident populations prior to this study. Invasion events in this study only occurred in populations of naïve translocated animals. Second, because resident populations had been exposed to pneumonia prior to radio-collaring, resident animals could only be categorized as naïve if born into a population during a healthy year. Animals remained naïve for each subsequent year that the population remained healthy. Inaccurate age estimates and failure to detect pneumonia within infected populations were likely to lead to misclassification of residents as naïve when in fact they were exposed. For these reasons, and the small sample size of translocated and resident animals that died of pneumonia in this study, the relationship between translocation and pneumonia risk seems to warrant further exploration.

### Limitations

Ideally, one would examine the relationship between exposure and immunity experimentally, by inoculating animals repeatedly at various intervals and following their fate, or by documenting individuals' serological status before and after pneumonia epidemics. However, the identity of the pathogen that causes pneumonia remains controversial, which poses a challenge for studies based on inoculation and serology. Given that field conditions such as weather and nutritional stress cannot be replicated in captivity [47]; that serological status is not necessarily correlated with protective immunity [48]; and that long-term re-exposure experiments are rarely feasible, we relied on population-level data to describe effects of exposure on individuals. As a result, a potential limitation of this study is misclassification. For example, we may have overestimated exposure if population substructuring (behavioral or spatial), or low transmission rates, led to incomplete exposure. Alternatively, we may have underestimated exposure if we failed to detect pneumonia outbreaks (a less likely scenario for ewes than rams, given that lambs provide a sentinel for pneumonia transmission in ewes). Assuming that some ewes were four years of age at capture also may have led to underestimation of exposure. Regardless of the direction of misclassification, in most cases the effect would be to increase similarity between the exposure history of the individual dying of pneumonia and the risk-set, therefore contributing to our inability to distinguish among hypotheses. A larger sample size of known-age adults, and adults that died of pneumonia, would have strengthened our analyses.

Another limitation of this study is difficulty differentiating between resistance to disease and resistance to infection. Animals that do not get sick may still be infected, or re-infected in subsequent epidemics (defined as ‘tolerant’ in some ecological literature) [49]. This distinction is important because chronically infectious animals, which are resistant to disease, will have profoundly different effects on the epidemiology of pneumonia than individuals that are resistant to infection and not infectious. The simultaneous presence of animals resistant to infection and animals that are carriers but protected from disease is consistent with observations of pneumonia in bighorn sheep in the wild and in captivity [25]–[28]. Carrier ewes within resistant populations are necessary to explain annual outbreaks in lambs in the absence of ewe mortality because lambs rarely contact other sources of pathogens (rams or domestic sheep) prior to weaning [24], [50]. Difficulty in differentiating between resistant and tolerant individuals may be common when using survival data to infer resistance to infection and is an important limitation of our study, given that a few tolerant individuals may be responsible for most disease transmission [51].

## Conclusions

By defining individuals' pneumonia-exposure histories, we tested hypotheses about immune response in a system for which immunological data are absent, the causative agent is unknown, and experimental approaches are not feasible. Without directly identifying the pathogen, we found that ewes develop some level of protective immunity following exposure; protection may wane in the absence of exposure or be boosted by repeated exposures; protective immunity is not effectively transferred from ewes to lambs; and unexposed animals translocated near infected populations have a high risk of developing pneumonia. Our results explain the high mortality during pathogen invasion and low adult mortality after invasion. The lack of protection via passive immunity in lambs suggests that pneumonia in bighorn sheep will lead to aging populations with limited recruitment. Although a larger sample size of animals that died of pneumonia would be desirable, most limitations stemmed from our inability to directly track a pathogen and therefore our inability to discriminate between resistant and tolerant (carrier) animals or to distinguish among epidemiological processes that might explain our findings. The recent discovery of *M. ovipneumoniae* as the probable primary pathogen provides further opportunities to test our hypotheses with additional field, laboratory, and dynamic modeling studies. We hope these studies will eventually inform development of management strategies that can break the cycle of prolonged pneumonia epidemics and aid recovery of bighorn sheep across their range.

## Supporting Information

Figure S1 **Data collection and pneumonia histories within Hells Canyon populations of bighorn sheep.** A. Individual pneumonia histories of 15 ewes within the Wenaha (population 2 in Fig. 2). Top panel: annual pneumonia status of the population based on a study-based time-scale. Bottom panel: annual pneumonia status of the population on an age-based time-scale. Red indicates years when adults and/or lambs died of pneumonia, green are years when no pneumonia mortality was detected (or suspected in lambs; see [20]); x's represent death or censoring. B. Annual pneumonia status in the 16 Hells Canyon bighorn sheep populations, 1994–2010 (see map in Fig. 2). 1 = Asotin, 2 = Wenaha, 3 = Mountain View, 4 = Black Butte, 5 = Redbird, 6 = Lower Hells Canyon, 7 = Imnaha, 8 = Big Canyon, 9 = Muir Creek, 10 = Meyers Creek, 11 = Saddle Creek, 12 = Upper Hells Canyon Oregon, 13 = Upper Hells Canyon Idaho, 14 = Sheep Mountain, 15 = Lostine, 16 = Bear Creek.
(PDF)

Figure S2 **Ewe pneumonia survival probability as a function of age.** The shaded area represents the 95% confidence bounds for the probability that a ewe of a given age died of pneumonia, using data included in the age-based proportional hazards models of ewe pneumonia mortality. Inclusion of an individual in each category of age is conditional on its survival up until that age, and each individual contributed as many data points as its age at last observation. The points are the proportion of ewes that survived to a given age-class and experienced a pneumonia epidemic that died of pneumonia during that epidemic.
(PDF)

Table S1 **Ewe relative risk of dying as a function of birth year pneumonia status for cementum-aged ewes.**
(DOCX)

Table S2 **Lambs: logistic regression results.**
(DOCX)

## References

[1] Hudson PJ, Rizzoli AR, Grenfell BT, Heesterbeek H, Dobson AP, editors (2002) The Ecology of Wildlife Diseases: Oxford University Press.

[2] Bolker B, Grenfell B (1995) Space, persistence and dynamics of measles epidemics. Philosophical Transactions of the Royal Society of London Series B-Biological Sciences 348: 309–320. 10.1098/rstb.1995.0070 8577828

[3] Bartlett MS (1957) Measles periodicity and community size. J R Statistical Soc A 120: 48–70.

[4] Grenfell BT, Bolker BM (1998) Cities and villages: infection hierarchies in a measles metapopulation. Ecology Letters 1: 63–70.

[5] Black S (1997) Epidemiology of pertussis. The Pediatric Infectious Disease Journal 16: S85–S89. 9109162 10.1097/00006454-199704001-00003

[6] Lavine JS, King AA, Bjornstad ON (2011) Natural immune boosting in pertussis dynamics and the potential for long-term vaccine failure. Proc Natl Acad Sci U S A 108: 7259–7264. 21422281 10.1073/pnas.1014394108 PMC3084147

[7] Güriş D, Strebel PM, Bardenheier B, Brennan M, Tachdjian R, et al (1999) Changing Epidemiology of Pertussis in the United States: Increasing Reported Incidence Among Adolescents and Adults, 1990–1996. Clinical Infectious Diseases 28: 1230–1237. 10451158 10.1086/514776

[8] Hudson PJ, Newborn D, Dobson AP (1992) Regulation and stability of a free-living host-parasite system: Trichostrongylus tenuis in red grouse: I. Monitoring and parasite reduction experiments. Journal of Animal Ecology 61: 477–486.

[9] Dobson AP, Hudson PJ (1992) Regulation and stability of a free-living host-parasite system: Trichostrongylus tenuis in red grouse: II. Population models. Journal of Animal Ecology 61: 487–498.

[10] Anderson R, May R (1991) Infectious Diseases of Humans. Oxford: Oxford Univ. Press.

[11] Besser TE, Cassirer EF, Yamada C, Potter KA, Herndon CN, et al (2012) Survival of bighorn sheep (*Ovis canadensis*) commingled with domestic sheep (*Ovis aries*) in the absence of *Mycoplasma ovipneumoniae* . Journal of Wildlife Disease 48. 10.7589/0090-3558-48.1.168 22247385

[12] Besser TE, Highland MA, Baker K, Cassirer EF, Anderson NJ, et al (2012) Causes of Pneumonia Epizootics among Bighorn Sheep, Western United States, 2008–2010. Emerg Infect Dis 18: 406–414. 22377321 10.3201/eid1803.111554 PMC3309594

[13] Besser TE, Cassirer EF, Potter KA, VanderSchalie J, Fischer A, et al (2008) Association of Mycoplasma ovipneumoniae infection with population-limiting respiratory disease in free-ranging Rocky Mountain bighorn sheep (*Ovis canadensis canadensis*). J Clin Microbiol 46: 423–430. 18057131 10.1128/JCM.01931-07 PMC2238132

[14] Cassirer EF, Sinclair ARE (2007) Dynamics of pneumonia in a bighorn sheep metapopulation. Journal Of Wildlife Management 71: 1080–1088.

[15] Singer FJ, Zeigenfuss LC, Spicer L (2001) Role of patch size, disease, and movement in rapid extinction of bighorn sheep. Conservation Biology 15: 1347–1354.

[16] Buechner HK (1960) The bighorn sheep in the United States, its past, present, and future. Wildlife Monographs 4: 3–174.

[17] Aune KE, Anderson N, Worley D, Stackhouse L, Henderson J, et al (1998) A comparison of population and health histories among seven Montana bighorn sheep populations. Proceedings Northern Wild Sheep and Goat Council 11: 46–69.

[18] Festa-Bianchet M (1988) A pneumonia epizootic in bighorn sheep, with comments on preventive management. Proceedings of the Biennial Symposium of the Northern Wild Sheep and Goat Council 6: 66–76.

[19] Jorgenson JT, Festa-Bianchet M, Gaillard J-M, Wishart WD (1997) Effects of age, sex, disease, and density on survival of bighorn sheep. Ecology 78: 1019–1032.

[20] Cassirer EF, Plowright RK, Manlove KR, Cross PC, Dobson AP, et al (2013) Spatio-temporal dynamics of pneumonia in bighorn sheep (*Ovis canadensis*). Journal of Animal Ecology DOI: 10.1111/1365-2656.12031. 10.1111/1365-2656.12031 23398603

[21] Coggins VL (1988) The Lostine Rocky Mountain bighorn sheep die-off and domestic sheep. Proceedings of the Biennial Symposium of the Northern Wild Sheep and Goat Council 6: 57–64.

[22] Foreyt WJ (1990) Pneumonia in bighorn sheep: effects of Pasteurella haemolytica from domestic sheep and effects on survival and long term reproduction. Proceedings of the Biennial Symposium of the Northern Wild Sheep and Goat Council 7: 92–101.

[23] Enk TA, Picton HD, Williams JS (2001) Factors limiting a bighorn sheep population in Montana following a dieoff. Northwest Science 75: 280–291.

[24] Festa-Bianchet M (1991) The social system of bighorn sheep: grouping patterns, kinship and female dominance rank. Animal Behaviour 42: 71–82.

[25] Miller MW, Hobbs NT, Williams ES (1991) Spontaneous pasteurellosis in captive Rocky Mountain bighorn sheep (*Ovis canadensis canadensis*): clinical, laboratory, and epizootiological observations. Journal of Wildlife Diseases 27: 534–542. 1758018 10.7589/0090-3558-27.4.534

[26] Sandoval AV, Elonowitz AS, Deforge JR (1987) Pneumonia in a transplanted population of bighorn sheep. Desert Bighorn Council Transactions 31: 18–22.

[27] Ward ACS, Hunter DL, Jaworski MD, Lane MV, Zaugg JL, et al (1992) Naturally occurring pneumonia in caesarian-derived Rocky Mountain bighorn sheep lambs. Biennial Symposium Northern Wild Sheep and Goat Council 8: 164–173.

[28] Foreyt WJ (1990) Pneumonia in bighorn sheep: effects of *Pasteurella haemolytica* from domestic sheep and effects on survival and long-term reproduction. Biennial Symposium Northern Wild Sheep and Goat Council: 92–101.

[29] Foster CL (2004) Wild Sheep Capture Guidelines. Proceedings Biennial Symposium Northern Wild Sheep and Goat Council 14: 211–282.

[30] Geist V (1966) Validity of horn segment counts in aging bighorn sheep. Journal of Wildlife Management 30: 634–646.

[31] Dimmick RW, Pelton MR (1994) Criteria of sex and age. In: Bookhout TA, editor. Research and management techniques for wildlife and habitats. Bethesda, Maryland, USA: The Wildlife Society. pp. 189–214.

[32] Therneau T (2011) A Package for Survival Analysis in S. R package version 2.36–12.

[33] Therneau T (2012) Mixed Effects Cox Models. R package version 2.2–3. Available: http://CRAN.R-project.org/package=coxme. Accessed 2013 March 30.

[34] Bates D, Maechler M, Bolker B (2011) lme4: Linear mixed-effects models using S4 classes. R package version 0.999375-42. Available: http://CRAN.R-project.org/package=lme4. Accessed 2013 March 30.

[35] R Development Core Team (2009) R: A language and environment for statistical computing. R Foundation for Statistical Computing, Vienna, Austria. ISBN 3-900051-07-0. Available: http://www.R-project.org.

[36] Therneau TM, Grambsch PM (2010) Modeling survival data: extending the Cox model. New York: Springer.

[37] Fieberg J, DelGiudice GD (2009) What time is it? Choice of time origin and scale in extended proportional hazards models. Ecology 90: 1687–1697. 19569383 10.1890/08-0724.1

[38] Festa-Bianchet M, King WJ (2007) Age–related reproductive effort in bighorn sheep ewes. Ecoscience 14: 318–322.

[39] Thernea T, Grambsch PM, Pankratz S (2003) Penalized survival models and frailty. Journal of computational and graphical statistics 12: 156–175.

[40] Shroff KE, Meslin K, Cebra JJ (1995) Commensal enteric bacteria engender a self-limiting humoral mucosal immune response while permanently colonizing the gut. Infection and immunity 63: 3904–3913. 7558298 10.1128/iai.63.10.3904-3913.1995 PMC173549

[41] Marcotte H, Lavoie MC (1998) Oral microbial ecology and the role of salivary immunoglobulin A. Microbiol Mol Biol Rev. 62: 71–109. 10.1128/mmbr.62.1.71-109.1998 PMC98907 9529888

[42] Vaupel JW, Manton KG, Stallard E (1979) The impact of heterogeneity in individual frailty on the dynamics of mortality. Demography 16: 439–454. 510638

[43] Hougaard P (1995) Frailty models for survival data. Lifetime Data Anal 1: 255–273. 9385105 10.1007/BF00985760

[44] Nunn CL, Lindenfors P, Pursall ER, Rolff J (2009) On sexual dimorphism in immune function. Philos Trans R Soc Lond B Biol Sci 364: 61–69. 18926977 10.1098/rstb.2008.0148 PMC2666693

[45] Moore SL, Wilson K (2002) Parasites as a Viability Cost of Sexual Selection in Natural Populations of Mammals. Science 297: 2015–2018. 12242433 10.1126/science.1074196

[46] Enk TA, Picton HD, Williams JS (2001) Factors limiting a bighorn sheep population in Montana following a dieoff. Northwest Science 75: 280–291.

[47] Plowright RK, Sokolow SH, Gorman ME, Daszak P, Foley JE (2008) Causal inference in disease ecology: investigating ecological drivers of disease emergence. Frontiers in Ecology and the Environment, DOI: 101890/070086 6 420–429.

[48] Gilbert A, Fooks AR, Hayman D, Horton DL, Müller T, et al.. (in revision) Deciphering serology to further understand the ecology of infectious diseases in wildlife. EcoHealth. 10.1007/s10393-013-0856-0 23918033

[49] Raberg L, Graham AL, Read AF (2009) Decomposing health: tolerance and resistance to parasites in animals. Philos Trans R Soc Lond B Biol Sci 364: 37–49. 18926971 10.1098/rstb.2008.0184 PMC2666700

[50] Bleich VC, Bowyer RT, Wehausen JD (1997) Sexual segregation in mountain sheep: Resources or predation? Wildlife Monographs 134: 1–50.

[51] Lloyd-Smith JO, Schreiber SJ, Kopp PE, Getz WM (2005) Superspreading and the effect of individual variation on disease emergence. Nature 438: 355–359. 16292310 10.1038/nature04153 PMC7094981

[52] Cassirer EF, Oldenburg LE, Coggins VL, Fowler P, Rudolph KM, et al (1996) Overview and Preliminary Analysis of a Bighorn Sheep Dieoff, Hells Canyon 1995–96. Biennial Symposium Northern Wild Sheep and Goat Council 10: 78–86.

