# Supplementary figures and images for: Use of Exposure History to Identify Patterns of Immunity to Pneumonia in Bighorn Sheep (Ovis canadensis)
Source: PLoS One. 2013 Apr 26;8(4):e61919. doi: 10.1371/journal.pone.0061919 (PMC3637318; doi:10.1371/journal.pone.0061919)

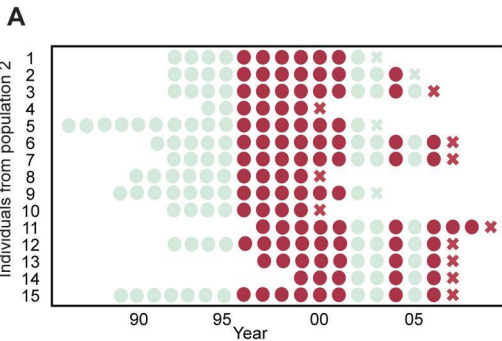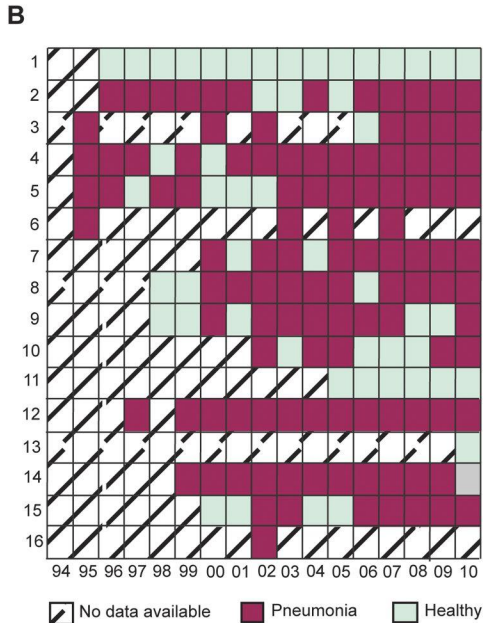

Supplement: Figure S1 — Data collection and pneumonia histories within Hells Canyon populations of bighorn sheep. A. Individual pneumonia histories of 15 ewes within the Wenaha (population 2 in Fig. 2). Top panel: annual pneumonia status of the population based on a study-based time-scale. Bottom panel: annual pneumonia status of the population on an age-based time-scale. Red indicates years when adults and/or lambs died of pneumonia, green are years when no pneumonia mortality was detected (or suspected in lambs; see [20]); x's represent death or censoring. B. Annual pneumonia status in the 16 Hells Canyon bighorn sheep populations, 1994–2010 (see map in Fig. 2). 1 = Asotin, 2 = Wenaha, 3 = Mountain View, 4 = Black Butte, 5 = Redbird, 6 = Lower Hells Canyon, 7 = Imnaha, 8 = Big Canyon, 9 = Muir Creek, 10 = Meyers Creek, 11 = Saddle Creek, 12 = Upper Hells Canyon Oregon, 13 = Upper Hells Canyon Idaho, 14 = Sheep Mountain, 15 = Lostine, 16 = Bear Creek. (PDF) [file pone.0061919.s001.pdf]

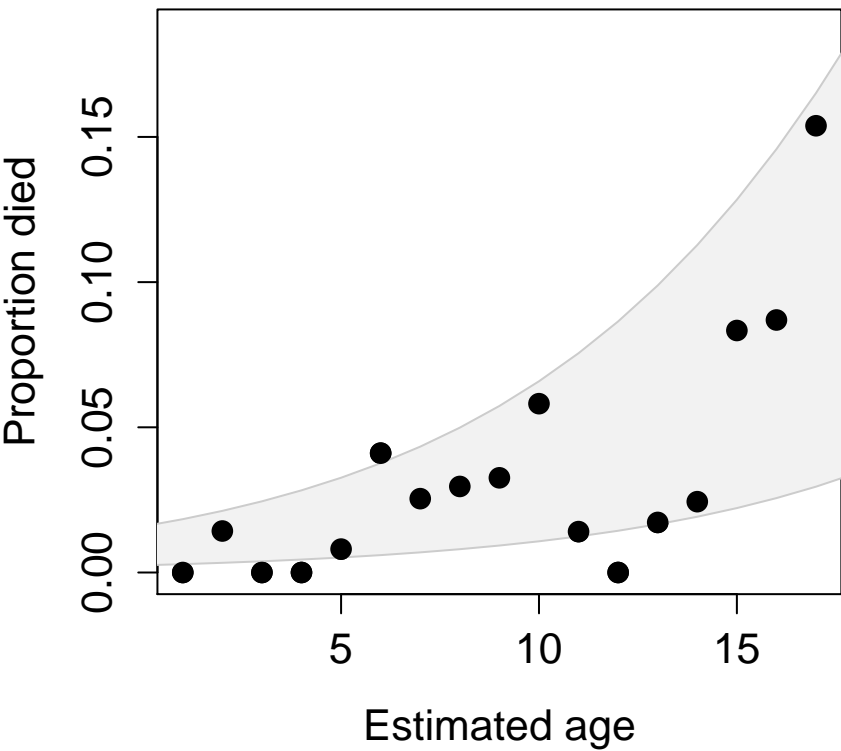

Supplement: Figure S2 — Ewe pneumonia survival probability as a function of age. The shaded area represents the 95% confidence bounds for the probability that a ewe of a given age died of pneumonia, using data included in the age-based proportional hazards models of ewe pneumonia mortality. Inclusion of an individual in each category of age is conditional on its survival up until that age, and each individual contributed as many data points as its age at last observation. The points are the proportion of ewes that survived to a given age-class and experienced a pneumonia epidemic that died of pneumonia during that epidemic. (PDF) [file pone.0061919.s002.pdf]
