# Supplementary material for: Use of Exposure History to Identify Patterns of Immunity to Pneumonia in Bighorn Sheep (Ovis canadensis)
Source: PLoS One. 2013 Apr 26;8(4):e61919. doi: 10.1371/journal.pone.0061919 (PMC3637318; doi:10.1371/journal.pone.0061919)
Supplement: Table S1 — Ewe relative risk of dying as a function of birth year pneumonia status for cementum-aged ewes. (DOCX) [file pone.0061919.s003.docx]

Table S1: Ewe relative risk of dying as a function of birth year pneumonia status for cementum-aged ewes

| **Model** | **Covariate** | **Beta** | **Exp. Beta (95% CI)** | **SE** | **P-value** | **AIC** |
| --- | --- | --- | --- | --- | --- | --- |
| PN Birth & Translocation | PN Birth | -0.04 | 0.96 (0.16, 12.91) | 0.83 | 0.96 | 57.7 |
|  | Translocated | 0.37 | 1.45 (0.16, 4.90) | 1.12 | 0.74 |  |
| PN Birth & Count & Translocation | PN Birth | -0.03 | 0.97 (0.19, 5.00) | 0.84 | 0.97 |  |
|  | Count | -0.01 | 0.99 (0.71, 1.38) | 0.17 | 0.95 | 59.7 |
|  | Translocated | 0.37 | 1.44 (0.16, 12.91) | 1.12 | 0.74 |  |

SE=Standard error; PN Birth=born in a year with a lamb pneumonia outbreak
