# Supplementary material for: Use of Exposure History to Identify Patterns of Immunity to Pneumonia in Bighorn Sheep (Ovis canadensis)
Source: PLoS One. 2013 Apr 26;8(4):e61919. doi: 10.1371/journal.pone.0061919 (PMC3637318; doi:10.1371/journal.pone.0061919)
Supplement: Table S2 — Lambs: logistic regression results. (DOCX) [file pone.0061919.s004.docx]

Table S2: Lambs: logistic regression results

| **Model** | **Covariates** | **Beta** | **Exp. Beta (95% CI)** | **P-value** | **AIC** |
| --- | --- | --- | --- | --- | --- |
| **Ewe only** | PN Year | 2.00 | 7.41 (5.39, 10.18) | <.0001 | 987.35 |
| **Count** | PN Year | 1.78 | 5.92 (4.22, 8.31) | <.0001 | 972.55 |
|  | Count | 0.14 | 1.15 (1.06, 1.23) | 0.00028 |  |
| **Translocation status** | PN Year | 1.98 | 7.24 (5.24, 9.99) | <.0001 |  |
|  | Translocated | -0.24 | 0.78 (0.52, 1.17) | 0.23 | 976.00 |
| **Age** | PN Year | 1.95 | 7.06 (5.12, 9.74) | <.0001 | 981.52 |
|  | Age Ewe | 0.08 | 1.08 (1.02, 1.14) | 0.0052 |  |
| **Age & Count** | PN Year | 1.79 | 6.02 (4.28, 8.45) | <.0001 |  |
|  | Age Ewe | 0.04 | 1.04 (0.97, 1.10) | 0.27 |  |
|  | Count | 0.11 | 1.12 (1.03, 1.22) | 0.0095 | 976.80 |

PN Year=year with pneumonia outbreak in lambs
